# Whole-Genome Sequence of *Mesorhizobium hungaricum* sp. nov. Strain UASWS1009, a Potential Resource for Agricultural and Environmental Uses

**DOI:** 10.1128/genomeA.01158-16

**Published:** 2016-10-13

**Authors:** Julien Crovadore, Bastien Cochard, Gautier Calmin, Romain Chablais, Torsten Schulz, François Lefort

**Affiliations:** a Plants and Pathogens Group, Research Institute Land Nature and Environment, hepia, HES-SO University of Applied Sciences and Arts Western Switzerland, Jussy, Geneva, Switzerland; b Faculty of Engineering and Architecture, HES-SO University of Applied Sciences and Arts Western Switzerland, Delémont, Switzerland; c Philotimo SA, Geneva, Switzerland

## Abstract

We report here the whole-genome shotgun sequences of the strain UASWS1009 of the species *Mesorhizobium hungaricum* sp. nov., which are different from any other known *Mesorhizobium* species. This is the first genome registered for this new species, which could be considered as a potential resource for agriculture and environmental uses.

## GENOME ANNOUNCEMENT

The genus *Mesorhizobium*, established in 1997, gathers rhizobacterial species genetically different from *Rhizobium* species (1). *Mesorhizobium* bacteria are mobile, aerobic, Gram-negative, and non-spore-forming rods and have a G+C content between 59 and 64% (2). These nitrogen-fixing bacteria may usually be found in symbiotic nodules or as endophytes in mimosoid temperate legumes (2). Strain UASWS1009 was isolated from the sewage sludge of a coking plant in Hungary through a selection experiment for highly ammonia-tolerant nitrifying bacteria. Initially identified as a *Mesorhizobium* species by 16S sequencing, it shared 98 to 99% identity with many *Mesorhizobium* strains in GenBank (3). Genomic DNA was extracted (4) and fragmented to an average size of 350 bp in a 50-µl Adaptive Focused Acoustics (AFA) microTUBE (Covaris, USA) in an S2 ultrasonicator (Covaris). The TruSeq DNA PCR-free library preparation kits (Illumina, USA) produced a library, of which insert sizes were checked in a fragment analyzer (Advanced Analytical Technologies, Inc.). Whole-genome shotgun (WGS) sequencing was performed in one Illumina MiniSeq run at 2 × 150-bp paired-end read length, using a MiniSeq Mid output kit (Illumina). The sequencing yielded 4,538,758 reads (680 Mb of DNA) providing 108-fold genome coverage. The reads are available from the NCBI Sequence Read Archive (SRA) database under the accession no. SRR4031082. Following quality control with FastQC (5) and assembly with SPAdes genome assembler 3.8.1 (6), the contigs were arranged with BioEdit (7) and analyzed with QUAST (8). The final assembly produced 41 contigs (≥200 bp), with a total genome length of 6,303,257 bp, a G+C content of 63.02%, and an *N*_50_ value of 631,149 bp. No plasmid was found by PlasmidFinder (9) and plasmidSPAdes (10). While RAST version 2.0 (11) annotated 6,111 coding sequence (CDS) genes distributed in 484 subsystems, the Prokaryotic Genome Automatic Annotation Pipeline (PGAAP) (12) identified 5,972 genes for 5,916 CDSs and 5,849 coding genes, 67 pseudogenes, and 56 RNA genes. A Mu prophage genome (30 genes) is integrated in contig 41. Mobile elements (22 sequences) are concentrated on contigs 22, 26, and 32. No virulence, disease, or toxin genes were found. It is equipped with genes for antibiotic production and resistance genes against metals (arsenic, cadmium, chrome, cobalt, copper, mercury, and zinc) and against a few antibiotics (penicillin, fluoroquinolones, streptothricin, and clavulanic acid). Diverse degradation pathways of aromatic compounds are provided by 80 genes, offering a potential use for environmental treatment of contaminated soils and water. Plant growth promotion properties are provided by genes for siderophore synthesis and transport (12 genes), 1-aminocyclopropane-1-carboxylate deaminase (ACC) for ethylene degradation (one gene), genes for plant hormones (13 genes), and antimicrobial compounds. Specific to plant-microbe associations, a large type IV secretion system of 35 genes was identified (13). The sequence similarity was found to be less than 80% with its closest phylogenetic neighbor *Mesorhizobium amorphae* strain CCNWGS0123 (14), which has a much larger genome. Contrary to this species, strain UASWS1009 had no *nif* and *nod* genes. As an environmental isolate, its legume hosts spectrum remains unknown. We propose the name *Mesorhizobium hungaricum* sp. nov. Crovadore and Lefort due to its geographical origin.

### Accession number(s).

This whole-genome shotgun (WGS) project was deposited at DDBJ/EMBL/GenBank under the accession no. MDEO00000000. The version described in this paper is the first version, MDEO00000000.1. The 41 contigs have been deposited under the accession numbers MDEO01000001 to MDEO01000041.
